# Neurocognitive effects of CSF biomarkers in idiopathic normal pressure hydrocephalus patients undergoing VP shunt placement

**DOI:** 10.1007/s10143-025-03609-8

**Published:** 2025-06-05

**Authors:** Hannah Spielmann, Magomed Lepshokov, Anna Prajsnar-Borak, Gudrun Wagenpfeil, Joachim Oertel

**Affiliations:** 1 Klinik für Neurochirurgie, Medizinische Fakultät, Universität des Saarlandes, Homburg, Saar Germany; 2 Institut für Medizinische Biometrie, Epidemiologie und Medizinische Informatik (IMBEI), Universität des Saarlandes, Fakultät für Medizin, Homburg, Saar Deutschland; 3 Klinik für Neurochirurgie, Landeskrankenhaus Feldkirch, Feldkirch, Österreich; 4 Klinik für Neurochirurgie, Universitätsklinikum des Saarlandes, Homburg, Saar Germany

**Keywords:** Hydrocephalus, Normal pressure hydrocephalus, Shunt therapy, Shunt valve adjustment, CSF biomarkers, Neurocognition

## Abstract

Idiopathic normal pressure hydrocephalus is an increasingly prevalent neurodegenerative condition among the elderly, characterized by the Hakim triad. Accurate diagnosis and prognosis are essential since the symptoms can be reversible with appropriate treatment, such as VP-shunt surgery. This study investigates the relationship between cerebrospinal fluid biomarkers, particularly Tau, Phospho-tau, the beta-amyloid ratio (Aβ42/Aβ40), and neurocognitive outcomes post-surgery. Eighty patients diagnosed with iNPH who underwent shunt placement between November 2021 and July 2023 were included. A comprehensive neuropsychological test battery was administered before, one hour after, and one day after lumbar puncture, and six weeks and three months post-surgery. CSF samples were analyzed for tau, phospho-tau and the beta-amyloid ratio. Neuropsychological tests assessed various cognitive functions, including executive functions, psychomotor speed, language, and memory. Patients with a higher beta-amyloid ratio showed significant cognitive improvement post-surgery. Neuropsychological tests, such as the DemTect and Trail Making Test A & B indicated enhanced performance over time, particularly at the three-month follow-up. Conversely, the MMSE did not show significant improvement. The data suggest that the beta-amyloid ratio is a potential prognostic marker for positive neurocognitive outcomes following VP-shunt surgery. The beta-amyloid ratio (Aß42/Aß40) may represent a valuable prognostic biomarker for predicting cognitive improvement after VP-shunt surgery. Patients with higher ratios exhibited better neurocognitive outcomes, emphasizing the importance of comprehensive neuropsychological assessments. These findings support the potential of personalized treatment strategies based on biomarker analysis to optimize patient selection and improve outcomes for iNPH patients undergoing VP-shunt surgery.

**Supplementary Information:**

The online version contains supplementary material available at 10.1007/s10143-025-03609-8.

## Clinical trial number

Not applicable.

**Supplementary Information:**

The online version contains supplementary material available at 10.1007/s10143-025-03609-8.

## Introduction

Idiopathic normal pressure hydrocephalus (iNPH) is an increasingly neurodegenerative condition in the aging population [13]. Characterized by the Hakim triad —gait disturbance, dementia, and urinary incontinence — iNPH presents a diagnostic and therapeutic challenge due to the variable expression of these symptoms [1]. Most frequently, iNPH is considered in differential diagnosis of dementia. Its correct diagnosis is even more important since iNPH symptoms can be reversible [12]. Thus, iNPH differs from almost all other forms of dementia in its rather favorable prognosis. Since treatment almost always includes shunt insertion, identifying suitable candidates is of utmost importance [32].

In other neurodegenerative diseases, such as Alzheimer’s disease (AD), cerebrospinal fluid (CSF) biomarkers are already established as vital diagnostic and prognostic tools. Biomarkers like the beta-amyloid ratio, the Tau protein and the Phospho-tau protein, provide critical insights into disease progression and treatment response in AD [2, 5]. Recent research has highlighted the beta-amyloid ratio (Aβ42/Aβ40) as a particularly important biomarker in diagnosing and understanding neurodegenerative diseases. The ratio’s interpretation depends on the concentrations of the Aβ42 and Aβ40 peptides in the CSF: a high or normal ratio suggests minimal deposition of Aβ42 in the brain, indicating a lower likelihood of neurodegeneration, whereas a low ratio points to pathological deposition and the formation of amyloid plaques, which are harmful to neurons [18–21, 24].

In iNPH, while many biomarkers have been investigated, their prognostic value remains ambiguous [5]. Some studies report a reduction in beta-amyloid levels in iNPH patients [10], while data on tau proteins are inconsistent, with some studies showing unchanged levels and others reporting increases [2, 10, 12]. Ideally, biomarkers in iNPH, could help predict patient outcomes. However, as of now, there are no established cut-off values or specific CSF markers for iNPH [18, 24].

The critical question remains whether these molecular markers, particularly when AD-specific protein characteristics are absent, can also predict treatment response, especially cognitive outcome, in iNPH patients?

## Methods

### Study design and participants

In the present study, we included 80 patients who underwent shunt placement between November 2021 and July 2023. All patients were diagnosed with iNPH. Patients with other neurological or neurodegenerative disorders that could affect cognitive function, such as AD, Parkinson’s disease, or vascular dementia, were excluded.

This study was approved by the local ethics committee (No. 147/20).

### Clinical protocol

All patients included in this study followed the same clinical protocol. The inclusion and exclusion criteria and the diagnostic work up was presented elsewhere in detail, please refer to Hülser et al. and Spielmann et al. for further information [11, 27].

In brief, patients underwent a thorough neurological examination. As part of this, gait assessment included the 10-meter walk test and a timed 360-degree turn, which were performed immediately prior to the neuropsychological test battery to establish a comprehensive functional baseline. Imaging diagnostics, including CT or MRI, were essential for the assessment, with MRI being preferred due to the absence of radiation exposure [12, 27, 32]. The diagnostic process followed the current guidelines of the German Society for Neurology and is consistent with the internationally recognized Relkin criteria [23]. Diagnosis was based on the presence of typical clinical symptoms such as gait disturbance, cognitive impairment, or urinary incontinence, in combination with characteristic imaging findings including an Evans Index greater than 0.3, enlarged inner ventricles, and signs of CSF distribution abnormalities [32].

If the combined radiological and clinical findings suggested a diagnosis of normal pressure hydrocephalus, the next step was a lumbar puncture. Before the lumbar puncture, a comprehensive neuropsychological test battery was conducted. These assessments served as a crucial baseline and comparison for all subsequent tests during the study.

### Lumbar puncture and CSF analysis

During the lumbar puncture, approximately 30–40 ml of cerebrospinal fluid (CSF) was drained into three tubes. The collected CSF was then sent to further laboratory analysis. The analysis included the examination of dementia markers, specifically beta-amyloid ratio (Aβ42/Aβ40), Tau- and Phospho-tau protein.

The biomarker assays used in this study were performed by the laboratory of Volkmann. For Tau protein, an ELISA kit from AJ Roboscreen GmbH (Leipzig, Germany) was used, with the code RE59631. For Phospho-tau, we used a Phospho-TAU ELISA assay from IBL (Hamburg, Germany), reference number 30,121,609. Beta-amyloid levels were measured using a CSF ELISA from IBL (Hamburg, Germany), code RE59651, from which the Aβ42/Aβ40 ratio was calculated. The reference values used for biomarker interpretation were as follows: Beta-Amyloid Ratio > 5.5% (normal), Tau < 445 pg/mL, and Phospho-Tau < 61 pg/mL. To exclude AD in our cohort, clinical follow-up and neuroimaging were conducted, and none of the patients exhibited the typical clinical features of AD, such as cognitive decline or amyloid plaques.

### Neuropsychological assessments

Patients underwent the neuropsychological test battery three times before shunt placement: before the lumbar puncture, one hour after, and one day after. If patients showed improvement, they were considered for VP-shunt placement. Further assessments were conducted six weeks and three months post-operation.

### Neuropsychological test battery

In this study, a comprehensive neuropsychological test battery was utilized to assess various items as recently published [12].

The Mini Mental Status Test (MMSE) evaluated cognitive performance and memory function, including attention, learning ability, and orientation. Scores range from 0 to 30, with scores below 9 indicating severe dementia and scores above 28 indicating no cognitive impairment [5]. The DemTect was employed to detect early stages of dementia, assessing executive functions, memory (immediate and delayed recall), language, and number recall. The maximum score is 18 points, with scores below 8 suggesting severe dementia and scores above 13 indicating age-appropriate cognitive function [17]. For evaluation of executive functions and psychomotor speed, the Stroop Test A and B was administered. In Part A, patients named the color of ink in which words were printed, while in Part B, they named the color of ink of incongruent color words. Time taken to complete each part was recorded [28]. The Digit Span Test A and B assessed short-term memory. In Part A, patients repeated sequences of numbers forwards, and in Part B, they repeated them backwards. The score reflects the longest sequence correctly recalled [8]. To assess psychomotor speed, mental flexibility, and executive functions, the Trail Making Test A and B was employed. In Part A, patients connected numbered circles sequentially, while in Part B, they alternated between numbers and letters in ascending order. Completion time for each part was recorded [25, 30, 31]. The Rey Auditory Verbal Learning Test was used to evaluate verbal learning and memory recall [9].

### Surgical protocol

All patients received a shunt set consisting of one adjustable differential pressure valve and one adjustable gravitational pressure valve plus an in line prechamber reservoir.

All patients underwent ventriculoperitoneal (VP) shunt placement under general anesthesia in the supine position, with proper positioning and padding. The head was fixed using a Mayfield clamp, and neuronavigation was established. Skin incisions were marked at the precoronal Kocher’s point, retroauricular region, and right paraumbilical area. Abdominal preparation included exposure of the peritoneum, placement of a purse-string suture, and temporary clamping of the peritoneum. A subcutaneous tunnel was created using a tunneling device from the abdomen to the retroauricular area.

A retroauricular pocket was prepared to accommodate the valve system consisting of the three parts. A submuscular channel was created to house the entire valve assembly securely.

Tunneling continued from the retroauricular incision to the precoronal entry point. At the precoronal site, a galea-periosteal flap was created to later seal the dural entry. A burr hole was placed under neuronavigational guidance. Following coagulation and incision of the dura and cortical surface, the right lateral ventricle was punctured using a Cushing needle and ventricular cannula.

At this step, intracranial pressure measurements were recorded, and cerebrospinal fluid (CSF) was sampled for dementia-related biomarker analysis. The Cushing needle was then removed, and a ventricular catheter was inserted 6 cm past the connector. A dural patch, from galea-periosteum, was placed around the catheter to ensure watertight sealing.

The shunt system was then connected and tunneled from the precoronal to the retroauricular site, and subsequently from the retroauricular site to the abdominal cavity. Valve function was confirmed intraoperatively by assessing filling and emptying of the pump chamber.

The peritoneum was opened, and the distal catheter was inserted and secured using the pre-placed purse-string suture. After a final functional check confirming continuous CSF flow, all surgical sites were closed in anatomical layers. Patients were extubated and transferred to the neurosurgical intensive care unit for postoperative monitoring.

### Statistical analysis

All statistical analysis were performed using SPSS v.29 (IBM, Armonk, USA). Results of the psychometric tests were coded as standardized z-scores according to the test norms of the respective manuals. X2, analysis of variance (ANOVA) as well as independent Student t test were used to compare the different groups. A value of *p* < 0.05 was considered statistically significant. A value of *p* < 0.10 was considered a statistical trend. Standard deviation is presented by ±. Range is presented in squared brackets.

All statistical analysis was performed with the support of a biostatistician. At first hand, patients were tried to be assigned to responders and non-responders. However, due to the multidimensional nature of the neuropsychological test battery and the heterogeneity of improvements across different cognitive domains, this classification proved inconsistent and methodologically unreliable. We also considered ROC analysis and logistic regression, but the scaling of the neuropsychological tests did not allow for their application. After consultation with the biostatistician (G.W.), patients were stratified into high and low biomarker expression groups, and group differences were analyzed using ANOVA and t-tests.

## Results

The total cohort comprised 80 individuals. Throughout the follow-up period, there were no fatalities, and all patients completed every scheduled follow-up assessment. All patients had normal beta-amyloid 42, normal tau and phospho-tau protein levels in the meaning of a non-AD specific pattern.

Patients were stratified into two groups based on the median value of tau, phospho-tau and beta-amyloid ratio into high and low expressing patients. The following section presents the neuropsychological findings.

In general, in MMSE, DemTect, Stroop Test A and B, and Digit Span Test A and B, higher Z-scores indicate better performance. Conversely, in Trail Making Test A and B, lower Z-scores indicate better performance.

### Neuropsychological results regarding the beta amyloid ratio (Aß42/Aß40)

The 80 patients in this study were stratified into two groups based on their median beta-amyloid ratio (Aß42/Aß40) levels: a low_(Aß42/Aß40)_ -group (*n* = 40) and a high_(Aß42/Aß40)_ -group (*n* = 40). The mean beta-amyloid ratio in the low group was 6.5 ± 1.3 pg/ml, while in the high group, it was 10.3 ± 2.1 pg/ml. As mentioned above, the (Aß42/Aß40) levels in the low- and high group were “healthy” in the sense of a non AD related value.

Preoperatively, there were no statistically significant differences between the low- and high _(Aß42/Aß40)_ -groups across any of the neuropsychological tests.

**Fig. 1:**
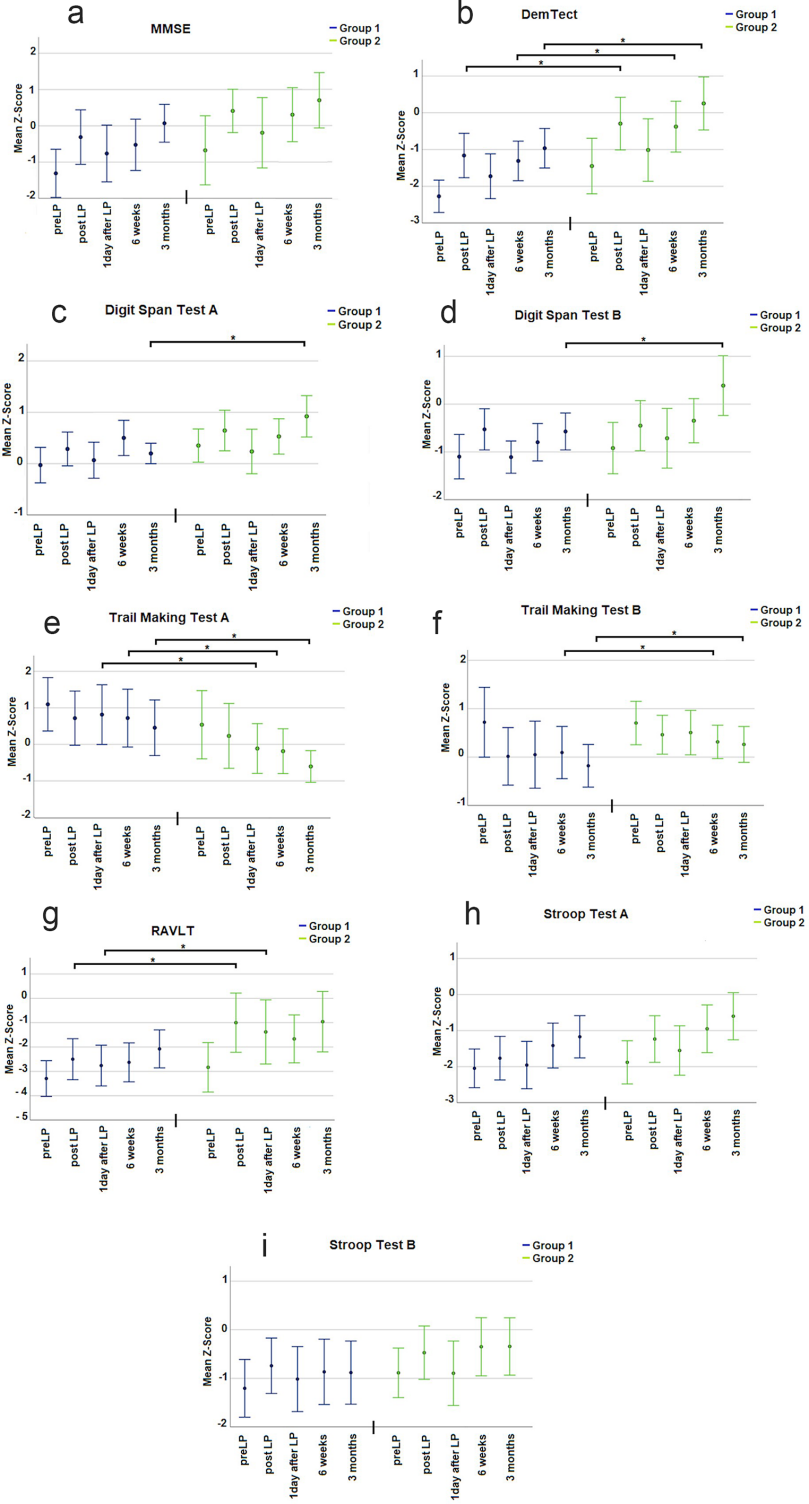
Results of the neuropsychological testing of the beta amyloid ratio (Aß42/40). Part A visualizes the results of the neuropsychological testing as z-score. In all sections on X-axis, different times of testing are depicted. Y-axis represents the z-score. The results of the low beta-amyloid ratio group are shown in blue, and the results of the high beta-amyloid ratio group are shown in green. Section **A** presents the results of MMSE. Section **B**, results of DemTect are shown. Section **C** presents the results of the Digit Span Test **A** and Section **D** shows the results of Digit Span Test **B**. Section **E** represents the results of the Trail Making Test **A** and Section **F** Trail Making Test **B**. RAVLT is shown in Section **G**. Section **H** visualizes the results of the Stroop Test **A** and Section **I** shows the results of the Stroop Test **B**

Three months after surgery patients of the high _(Aß42/Aß40)_ -group performed significantly better in DemTect (B), in Digit Span Test A (C) and B (D) and Trail Making Test A (E) and B (F) compared to patients of the low _(Aß42/Aß40)_ -group. Furthermore was at the same postoperative follow-up in MMSE (A), Stroop Test A (H) and RAVLT (G) a trend observable for a higher neurocognitive performance in patients with a high _(Aß42/Aß40)_-ratio. For additional details, please refer to Tables 1, 2 and 3; Fig. 1.

### Neuropsychological results regarding Tau

The 80 patients were divided into two groups based on their median tau protein expression: a low _(tau)_ -group (*n* = 40) and a high _(tau)_ -group (*n* = 40). The mean tau protein level was 104.8 ± 7.8 pg/ml in the low _(tau)_ -group and 641.6 ± 7.1 pg/ml in the high _(tau)_ -group. Emphasize has to be given to the fact that all tau values were non pathologic in an AD related sense.

Preoperatively, there were no statistically significant differences between the low and high tau groups across any of the neuropsychological tests.

In the dementia assessments, including MMSE and DemTect, no significant differences were observed between the two groups at any time. Similarly, no significant differences were found in any of the other neuropsychological tests at the three-month follow-up.

For additional details, please refer to Table 4.

### Neuropsychological results regarding Phospho-Tau

The 80 patients were divided into two groups based on their median phospho-tau protein expression: a low _(phospho−tau)_ -group (*n* = 40) and a high _(phospho−tau)_ -group (*n* = 40). The mean phospho-tau protein level was 29.3 ± 6.7 pg/ml in the low _(phospho−tau)_ - group and 58.2 ± 30.8 pg/ml in the high _(phospho−tau)_- group, these values were again non-AD related.

Preoperatively, there were no statistically significant differences between the low and high phospho-tau groups across any of the neuropsychological tests except RAVLT. In RAVLT patients with a high _(phospho−tau)_ expression performed significantly better.

In the dementia assessments, including the DemTect and MMSE, no significant differences were found at any testing time. Similar can be stated for Trail Making Test A and B, Stroop Test A and B, Digit Span Test B with no significant differences between the low _(phospho−tau)_ and the high _(phospho−tau)_ -group at any testing time. In the Stroop Test B, a significant difference between both groups for a better performance in the high _(phospho−tau)_- group was found immediately after the lumbar puncture, but this was not sustained at later testing times.

In the Digit Span Test A, however, patients showed significant improvement at the three-months follow-up. The better pre-lumbar puncture performance of patients in the high _(phospho−tau)_ -group in RAVLT remained observable up to three months post-operatively. For additional details, please refer to Table 5.

### Complication management protocol and postoperative pressure adjustment

No intraoperative or early postoperative complications such as hemorrhage, wound healing disorders, or malpositioning of shunt components were observed. No planned shunt revisions were necessary in the immediate postoperative period. Valve pressure adjustments were not routinely performed postoperatively but remained possible as needed during follow-up to optimize individual clinical outcomes. A shunt adjustment was made in 6 patients within the follow up period of three months since no effect of shunt insertion was reported and found. In all of them, the standard settings of ProGav 8 cm H2O and Mblue 28 cm H2O were adjusted to 6/28.

## Discussion

Our study aimed to investigate the relationship between various dementia markers- namely the beta-amyloid ratio, Tau protein, and Phospho-Tau protein in CSF and neurocognitive outcomes in patients with iNPH following VP-shunt surgery. These molecular markers are widely recognized for their role in identifying and predicting various neurodegenerative diseases [3, 7, 16]. It is therefore logical to hypothesize that these CSF proteins could also serve as predictive markers for cognitive outcomes in iNPH patients. Furthermore, their analytical measurement is standardized in neurological laboratories worldwide.

It is important to clarify that the combination of biomarkers typically associated with AD was not present in our study. While elevated tau levels in the high group (641.6 pg/ml) exceed the pathological cut-off of 445 pg/ml, this elevation does not necessarily indicate AD pathology. Potential explanations for the increased Tau levels include heightened cerebrospinal fluid turnover and mechanical stress, both of which can lead to elevated tau without suggesting underlying AD [2]. Additionally, none of the patients in our cohort met the clinical criteria for AD, and AD was explicitly excluded based on clinical evaluation and neuroimaging. Therefore, the elevated tau levels observed in our study are most likely not linked with AD.

It has to be noted that under the query of the paper at hand the results of these measurements has to be scrutinized carefully: The established cut-off values for these protein expressions are based on studies of different neurodegenerative diseases, primarily AD, and are not specific to iNPH [26]. It has therefore to be emphasized that all patients in the cohort at hand had in that sense normal or “healthy” values. This suggests that the pathological processes typically seen in neurodegenerative diseases like AD, such as amyloid plaque formation and tau protein aggregation, were not prominent in our cohort. This distinction is essential as it supports the hypothesis that iNPH, unlike AD, can potentially be reversed with appropriate treatment such as VP-shunt surgery. So far literature is not able to provide specific iNPH related and generally accepted liquor markers or cut-off values [26].

One of the distinctive features of our study is the extensive and detailed neuropsychological assessment conducted. Previous research has primarily focused on gait disturbance on one hand or for example MMSE on the other hand when capturing postoperative performance and neurocognition. Only few other author give here a more comprehensive picture of the neurological reality [8–10, 29].

In the manuscript at hand the neurocognitive function of patients was evaluated in a holistic manner addressing various aspects of neurocognition providing a more nuanced understanding of the cognitive changes in iNPH patients following lumbar puncture and VP-shunt surgery.

The data at hand indicates that especially beta-amyloid ratio (Aß42/Aß40) could be a potential candidate for predicting neurocognitive therapy response to VP shunting in iNPH patients. Patients with a higher beta-amyloid ratio (high_(Aß42/Aß40)_ -group) demonstrated better cognitive outcomes post-surgery than those of the low_(Aß42/Aß40)_ -group up to three months postoperatively. The initial performance levels in various neurocognitive testing before lumbar puncture was not distinguishable between the two groups. This indicates a specific value of the beta-amyloid ratio (Aβ42/Aβ40) to predict postoperative neurocognitive outcome after shunt placement. The picture was not that clear in the other two scrutinized liquor proteins Tau and Phospho-Tau. The first one could not demonstrate any postoperative difference in neurocognitive testing between patients with low and high Tau liquor values. The later, Phospho-Tau, demonstrated in two neurocognitive tests a better performance in the high _(phospho−tau)_- group postoperatively. It has to be noted that at least in RAVLT already before lumbar puncture a higher score in _low/high_ expressing Phospho-Tau patients was observable. Therefore, no reliable conclusion should be drawn regarding its value as predictive marker.

Some studies have already demonstrated the disturbance of CSF dynamics and dysmetabolism in iNPH patients [6]. For example the beta-amyloid Aβ42 expression in CSF may influence gait outcomes [4]. Jeppsson et al. demonstrated that the combination of t-tau, Aß40, and MCP-1 separates iNPH patients from other cognitive and movement disorders, but the focus was not on predictive neurocognitive outcomes or even aspects of the Hakim Triad [15]. Lukkarinen et al. also showed the relationship between CSF biomarkers and clinical features in iNPH patients, but only using the MMSE and gait testing and were thus not giving a comprehensive approach to neurocognition [22]. To the best knowledge of the authors was the finding that a high beta-amyloid ratio (Aβ42/Aβ40) may be correlated with better treatment response to VP shunting not been published before [22].

Our findings suggest that a higher Aβ42/Aβ40 ratio is associated with better postoperative cognitive outcomes in iNPH patients. One possible explanation is that a higher ratio indicates a lower likelihood of comorbid AD pathology. In line with this, previous studies have demonstrated that the presence of AD pathology, as reflected by lower Aβ42/Aβ40 ratios, is associated with reduced shunt responsiveness and poorer cognitive recovery [14].

Thus, the Aβ42/Aβ40 ratio may serve as a proxy for cerebral plasticity or cognitive reserve in the iNPH population and could potentially help to stratify patients regarding their expected benefit from surgery. However, our results should be interpreted cautiously, as the direct relationship between Aβ pathology and surgical outcome remains complex and may be influenced by multiple additional factors [18, 20, 24].

The observed postoperative improvement in cognitive performance is likely multifactorial. One contributing factor may be the reduction of intracranial pressure and ventricular distension following shunt placement, which could lead to a reversal of periventricular ischemia and decreased mechanical stress on adjacent brain structures. These effects may facilitate functional recovery in brain regions involved in cognition, such as the frontal and subcortical circuits commonly affected in iNPH [14].

### Limitations and conclusion

The authors acknowledge that the follow-up period was limited to three months post-surgery, which may not capture long-term cognitive outcomes and the potential for symptom recurrence. Longer follow-up periods are needed to fully understand the durability of the observed cognitive improvements.

The use of the beta-amyloid ratio as a prognostic tool opens new avenues for personalized medicine in iNPH. By stratifying patients based on their beta-amyloid ratio, clinicians can better predict which patients are likely to benefit most from VP-shunt surgery. This approach can improve patient outcomes, reduce unnecessary procedures, and optimize healthcare resources.

Another important limitation is the lack of postoperative CSF biomarker data. While preoperative levels of Aβ42/Aβ40, tau, and phospho-tau were assessed, no postoperative measurements were taken. As a result, we were unable to evaluate the dynamic changes of these biomarkers after shunt placement or their direct relationship to cognitive recovery. Future studies incorporating postoperative CSF sampling will be essential to further elucidate the role of biomarker trajectories in predicting and monitoring treatment response in iNPH.

Future research should focus on long-term follow-up studies to understand the durability of cognitive improvements and the potential for symptom recurrence. Additionally, investigating the interaction between other CSF biomarkers and the beta-amyloid ratio could provide a more comprehensive understanding of the pathophysiology of iNPH and its treatment outcomes.

## Electronic supplementary material

Below is the link to the electronic supplementary material.

Supplementary Material 1

Supplementary Material 2

Supplementary Material 3

Supplementary Material 4

## Data Availability

No datasets were generated or analysed during the current study.

## Abbreviations

AD: Alzheimer’s disease
CSF: Cerebrospinal fluid
CT: Computertomography
DemTect: Dementia Detection Test
iNPH: Idiopathic normal pressure hydrocephalus
MMSE: Mini Mental State Examination Test
MRI: Magnet Resonance Imaging
RAVLT: Rey Auditory Verbal Learning Test
VP-shunt: Ventriculoperitoneal shunt
