# Supplementary material for: Neurocognitive effects of CSF biomarkers in idiopathic normal pressure hydrocephalus patients undergoing VP shunt placement
Source: Neurosurg Rev. 2025 Jun 5;48(1):484. doi: 10.1007/s10143-025-03609-8 (PMC12141128; doi:10.1007/s10143-025-03609-8)
Supplement: Supplementary file 1 — Supplementary Material 1 [file 10143_2025_3609_MOESM1_ESM.docx]

Table 1: Neuropsychological results of the beta amyloid ratio, p-value, low group vs. high group

| Test | before lp | after lp | 1 day after lp | 6 weeks | 3 months |
| --- | --- | --- | --- | --- | --- |
| MMSE | *p* = 0.140 | *p* = 0.068 | *p* = 0.182 | *p* = 0.055 | *p* = 0.082 |
| DemTect | *p* = 0.055 | *p* = 0.033 | *p* = 0.082 | *p* = 0.017 | *p* = 0.004 |
| Digit Span A | *p* = 0.055 | *p* = 0.081 | *p* = 0.268 | *p* = 0.453 | *p* = 0.001 |
| Digit Span B | *p* = 0.314 | *p* = 0.425 | *p* = 0.130 | *p* = 0.068 | *p* = 0.004 |
| Trail Making Test A | *p* = 0.172 | *p* = 0.200 | *p* = 0.045 | *p* = 0.036 | *p* = 0.013 |
| Trail Making Test B | *p* = 0.242 | *p* = 0.044 | *p* = 0.021 | *p* = 0.001 | *p* = 0.008 |
| RAVLT | *p* = 0.230 | *p* = 0.023 | *p* = 0.041 | *p* = 0.065 | *p* = 0.062 |
|  |  |  |  |  |  |
| Stroop Test A | *p* = 0.388 | *p* = 0.115 | *p* = 0.196 | *p* = 0.152 | *p* = 0.097 |
| Stroop Test B | *p* = 0.206 | *p* = 0.248 | *p* = 0.399 | *p* = 0.125 | *p* = 0.111 |
