# Supplementary material for: Neurocognitive effects of CSF biomarkers in idiopathic normal pressure hydrocephalus patients undergoing VP shunt placement
Source: Neurosurg Rev. 2025 Jun 5;48(1):484. doi: 10.1007/s10143-025-03609-8 (PMC12141128; doi:10.1007/s10143-025-03609-8)
Supplement: Supplementary file 2 — Supplementary Material 2 [file 10143_2025_3609_MOESM2_ESM.docx]

Table 2: results of the beta amyloid ratio, low group, mean points

| test | before lp | after lp | 1 day after lp | 6 weeks | 3 months |  |
| --- | --- | --- | --- | --- | --- | --- |
| MMSE | -1.1 ± 0.4 | -0.3 ± 0.3 | -0.8 ± 0.4 | -0.5 ± 0.3 | 0.1 ± 0.2 |  |
| DemTect | -2.0 ± 0.2 | -1.2 ± 0.3 | -1.7 ± 0.3 | -1.3 ± 0.26 | -1.0 ± 0.3 |  |
| Digit Span A | 0.0 ± 0.2 | 0.3 ± 0.2 | 0.1 ± 0.2 | 0.5 ± 0.2 | 0.2 ± 0.1 |  |
| Digit Span B | -1.1± 0.3 | -0.5 ± 0.2 | -1.1± 0.2 | -0.8 ± 0.2 | -0.6 ± 0.2 |  |
| Trail Making Test A | 1.1 ± 0.4 | 0.7 ± 0.4 | 0.8 ± 0.4 | 0.7 ±0.4 | 0.4 ± 0.4 |  |
| Trail Making Test B | -0.1 ± 0.3 | 0.1 ± 0.3 | 0.1 ± 0.4 | 0.1 ± 0.3 | -0.3 ± 0.2 |  |
| RAVLT | | -3.2 ± 0.3 | -2.5 ± 0.4 | -2.7 ± 0.4 | -2.6 ± 0.4 | -2.0 ± 0.4 |
| Stroop Test A | -2.0 ± 0.3 | -1.7 ± 0.3 | -2.0 ± 0.4 | -1.4 ± 0.3 | -1.2 ± 0.3 |  |
| Stroop Test B | -1.2 ± 0.3 | -0.7 ± 0.3 | -1.0 ± 0.3 | -0.9 ± 0.3 | -0.9 ± 0.3 |  |

Table 3: results of the beta amyloid ratio, high group, mean points

| test | before lp | after lp | 1 day after lp | 6 weeks | 3 months |
| --- | --- | --- | --- | --- | --- |
| MMSE | -0.9 ± 0.4 | 0.4 ± 0.2 | -0.2 ± 0.5 | 0.3 ± 0.4 | 0.7 ± 0.4 |
| DemTect | -1.6 ± 0.4 | -0.3 ± 0.4 | -1.0 ± 0.4 | -0.4 ± 0.3 | 0.2 ± 0.3 |
| Digit Span A | 0.4 ± 0.2 | 0.7 ± 0.2 | 0.3 ± 0.2 | 0.5 ± 0.2 | 0.9 ± 0.2 |
| Digit Span B | -0.9 ± 0.26 | -0.5± 0.3 | -0.7 ± 0.3 | -0.4 ± 0.2 | 0.4 ± 0.4 |
| Trail Making Test A | 0.5 ± 0.5 | 0.2 ± 0.4 | -0.1 ± 0.3 | - 0.2 ± 0.3 | -0.6 ± 0.2 |
| Trail Making Test B | -0.2 ± 0.2 | -0.5 ± 0.2 | -0.4± 0.3 | -0.7 ± 0.2 | -0.7 ± 0.3 |
| RAVLT | -2.8 ± 0.5 | -1.0 ± 0.5 | -1.3 ± 0.6 | -1.7 ± 0.4 | -1.0 ± 0.6 |
| Stroop Test A | -1.8 ± 0.3 | -1.2 ± 0.3 | -1.5 ± 0.3 | -0.9 ± 0.3 | -0.6 ± 0.3 |
| Stroop Test B | -0.9 ± 0.2 | -0.5 ± 0.3 | -0.9 ± 0.3 | -0.3 ± 0.3 | -0.3 ± 0.3 |
