# Supplementary material for: Neurocognitive effects of CSF biomarkers in idiopathic normal pressure hydrocephalus patients undergoing VP shunt placement
Source: Neurosurg Rev. 2025 Jun 5;48(1):484. doi: 10.1007/s10143-025-03609-8 (PMC12141128; doi:10.1007/s10143-025-03609-8)
Supplement: Supplementary file 3 — Supplementary Material 3 [file 10143_2025_3609_MOESM3_ESM.docx]

Table 4: Neuropsychological results of the Tau protein, p-value, low group vs. high group

| Test | before lp | after lp | 1 day after lp | 6 weeks | 3 months |
| --- | --- | --- | --- | --- | --- |
| MMSE | *p* = 0.117 | *p* = 0.310 | *p* = 0.124 | *p* = 0.053 | *p* = 0.080 |
| DemTect | *p* = 0.234 | *p* = 0.284 | *p* = 0.075 | *p* = 0.040 | *p* = 0.290 |
| Digit Span A | *p* = 0.176 | *p* = 0.111 | *p* = 0.085 | *p* = 0.383 | *p* = 0.111 |
| Digit Span B | *p* = 0.052 | *p* = 0.017 | *p* = 0.064 | *p* = 0.077 | *p* = 0.118 |
| Stroop Test A | *p* = 0.262 | *p* = 0.372 | *p* = 0.285 | *p* = 0.298 | *p* = 0.398 |
| Stroop Test B | *p* = 0.205 | *p* = 0.230 | *p* = 0.494 | *p* = 0.350 | *p* = 0.227 |
| RAVLT | *p* = 0.167 | *p* = 0.064 | *p* = 0.036 | *p* = 0.013 | *p* = 0.055 |
| Trail Making Test A | *p* = 0.495 | *p* = 0.186 | *p* = 0.010 | *p* = 0.247 | *p* = 0.075 |
| Trail Making Test B | *p* = 0.265 | *p* = 0.310 | *p* = 0.433 | *p* = 0.228 | *p* = 0.330 |
