# Supplementary material for: Neurocognitive effects of CSF biomarkers in idiopathic normal pressure hydrocephalus patients undergoing VP shunt placement
Source: Neurosurg Rev. 2025 Jun 5;48(1):484. doi: 10.1007/s10143-025-03609-8 (PMC12141128; doi:10.1007/s10143-025-03609-8)
Supplement: Supplementary file 4 — Supplementary Material 4 [file 10143_2025_3609_MOESM4_ESM.docx]

Table 5: Neuropsychological results of the phospho-tau protein, p-value, low group vs. high group

| Test | before lp | after lp | 1 day after lp | 6 weeks | 3 months |
| --- | --- | --- | --- | --- | --- |
| MMSE | *p* = 0.364 | *p* = 0.060 | *p* = 0.498 | *p* = 0.247 | *p* = 0.255 |
| DemTect | *p* = 0.458 | *p* = 0.458 | *p* = 0.222 | *p* = 0.295 | *p* = 0.209 |
| Digit Span A | *p* = 0.277 | *p* = 0.267 | *p* = 0.315 | *p* = 0.322 | *p* = 0.035 |
| Digit Span B | *p* = 0.200 | *p* = 0.224 | *p* = 0.416 | *p* = 0.069 | *p* = 0.110 |
| Stroop Test A | *p* = 0.260 | *p* = 0.280 | *p* = 0.368 | *p* = 0.343 | *p* = 0.260 |
| Stroop Test B | *p* = 0.087 | *p* = 0.031 | *p* = 0.250 | *p* = 0.236 | *p* = 0.149 |
| RAVLT | *p* = 0.024 | *p* = 0.110 | *p* = 0.048 | *p* = 0.100 | *p* = 0.043 |
| Trail Making Test A | *p* = 0.459 | *p* = 0.221 | *p* = 0.027 | *p* = 0.338 | *p* = 0.055 |
| Trail Making Test B | *p* = 0.229 | *p* = 0.137 | *p* = 0.271 | *p* = 0.115 | *p* = 0.406 |
